# Two-way pharmacodynamic modeling of drug combinations and its application to pairs of repurposed Ebola and SARS-CoV-2 agents

**DOI:** 10.1128/aac.01015-23

**Published:** 2024-03-12

**Authors:** Shuang Xu, Shadisadat Esmaeili, E. Fabian Cardozo-Ojeda, Ashish Goyal, Judith M. White, Stephen J. Polyak, Joshua T. Schiffer

**Affiliations:** 1 Fred Hutchinson Cancer Research Center, Vaccine and Infectious Diseases Division, Seattle, Washington, USA; 2 Department of Microbiology, University of Virginia, Charlottesville, Virginia, USA; 3 Department of Cell Biology, University of Virginia, Charlottesville, Virginia, USA; 4 Virology Division, Department of Laboratory Medicine and Pathology, University of Washington, Seattle, Washington, USA; 5 Department of Global Health, University of Washington, Seattle, Washington, USA; 6 Department of Microbiology, University of Washington, Seattle, Washington, USA; 7 Division of Allergy and Infectious Diseases, University of Washington, Seattle, Washington, USA; Providence Portland Medical Center, Portland, Oregon, USA

**Keywords:** two-way pharmacodynamic modeling, dose-response matrix, concentration-dependent drug-drug interaction, antiviral drug combinations, upstream drug-dependent pharmacodynamic parameters, drug repurposing

## Abstract

Existing pharmacodynamic (PD) mathematical models for drug combinations discriminate antagonistic, additive, multiplicative, and synergistic effects, but fail to consider how concentration-dependent drug interaction effects may vary across an entire dose-response matrix. We developed a two-way pharmacodynamic (TWPD) model to capture the PD of two-drug combinations. TWPD captures interactions between upstream and downstream drugs that act on different stages of viral replication, by quantifying upstream drug efficacy and concentration-dependent effects on downstream drug pharmacodynamic parameters. We applied TWPD to previously published *in vitro* drug matrixes for repurposed potential anti-Ebola and anti-SARS-CoV-2 drug pairs. Depending on the drug pairing, the model recapitulated combined efficacies as or more accurately than existing models and can be used to infer efficacy at untested drug concentrations. TWPD fits the data slightly better in one direction for all drug pairs, meaning that we can tentatively infer the upstream drug. Based on its high accuracy, TWPD could be used in concert with PK models to estimate the therapeutic effects of drug pairs *in vivo*.

## INTRODUCTION

Drug combinations are standard for several viral diseases in humans, including human immunodeficient viruses (HIV) and hepatitis C virus (HCV) (1, 2). In addition to diminishing the risk of drug resistance, drug synergy may allow therapeutic antiviral effects while minimizing side effects caused by higher drug concentrations required for monotherapies (3–6). Drug combinations may have a role in the treatment of emerging viral diseases with pandemic potential that lack effective prophylactic or therapeutic measures (7–11). Of particular interest is combining repurposed, licensed agents that lack sufficient antiviral potency on their own. Screening repurposed drug combinations against novel pathogens is less costly and more rapid compared to the *de novo* development of sequence-dependent countermeasures like antibodies and customized directly acting antiviral agents. Therefore, combinations of repurposed antivirals may have high utility early during a pandemic.

High throughput multidose matrix assays are widely used to identify synergistic drug combinations with antiviral activity against Zika (12, 13), arenaviruses (14), Ebola (4, 15), and SARS-CoV-2 (16–19). The results of those assays are reported in dose-response matrixes, with the efficacy of each combination reported within each matrix locus. To identify synergistic combinations based on the dose-response matrixes, there are available tools, such as SynergyFinder 3.0 (20–22) and MacSynergy II (23, 24), which calculate synergy scores based on deviation in observed combined effects from expected efficacy of a drug combination given by different reference models, including Highest Single Agent (HSA) (25), Loewe additivity (26), Bliss independence (27), and Zero interaction potency (ZIP) (28). Loewe additivity assumes drugs work via an equivalent mechanism with competition for the same binding site, while in our case, Bliss independence assumes that drugs work on different steps of the viral replication cycle generating multiplicative effects. Based on which reference model best explains the data, drug-drug interactions can be classified as antagonistic, Loewe additive, intermediate, Bliss independent, or synergistic, among which synergy has the highest increase in combined efficacy, exceeding that predicted by multiplicative models (29). The quantified synergy score demonstrates the strength of drug-drug interaction and the measured biological effect.

The degree of synergy is often variable across different drug concentrations (30). The combined efficacy may be increased in highly synergistic areas of a dose-response matrix, with less of an increase or even a decrease in other matrix regions. The accuracy of PD modeling of a combination relies on closely capturing concentration-dependent drug-drug interactions. Because drug levels fluctuate over time in humans according to the drug’s pharmacokinetic (PK) characteristics, a useful model must accurately predict the efficacy of combined therapies over a wide range of dose combinations, including those not measured experimentally, to optimize preclinical testing and clinical trials (31–38).

PD reference models such as Loewe additivity and Bliss independence may fail to capture the full complexity of concentration-dependent drug-drug interactions because they may only be applicable to certain areas within the drug combination matrix. Response-surface-based approaches, which assume Loewe additivity or Bliss independence, only partially capture concentration dependencies in drug-drug interactions (39–41). The general pharmacodynamic interaction (GPDI) model was built upon subpopulation synergy models (35, 42–44) and evaluates a global interaction term to model the efficacy of drug combinations with flexibility in interaction assumptions. Additionally, it examines the directionality of drug-drug interaction by looking at how one drug affects the PD parameters of another and vice versa (30). A possible limitation of GPDI is that a global interaction term over the dose-response matrix may not accurately recapitulate experimental PD data because of nonlinearity of concentration-dependent interaction indexes (45). The zero interaction potency (ZIP) model follows a similar logic as it calculates expected efficacy with unaltered PD parameters of the downstream drug by another drug and quantifies delta scores based on observed efficacy and expectations for the interaction landscape surface (28). ZIP uses the interaction landscape surface to identify synergistic combinations but is not equipped to mathematically predict the efficacy of all possible drug concentration combinations (28), including those not measured experimentally.

We developed a two-way pharmacodynamic (TWPD) modeling approach to capture concentration-dependent drug-drug interactions and accurately recapitulate the efficacy of drug combinations over the entire dose-response matrix. We also studied the directionality of drug-drug interactions (i.e., two-way pharmacodynamics) by switching the role (upstream or downstream of where in the virus lifecycle the drug acts) of drugs and comparing the accuracy of the model under these competing scenarios. We tested the predictive power of TWPD by applying the approach to two previously published repurposed anti-Ebola drug combination *in vitro* data sets (sertraline + bepridil and sertraline + toremifene) (4), among which bepridil, sertraline, and toremifene are used for hypertension, depression, and breast cancer, respectively (46, 47). We also applied TWPD to two previously published repurposed anti-SARS-CoV-2 drug combination data sets (nitazoxanide + remdesivir and nitazoxanide + arbidol)(16), the components of which include two broad-spectrum antiviral drugs [arbidol (48, 49) and remdesivir (50–52)] and an antiprotozoal agent [nitazoxanide (53)]. In both cases, *in vitro* assessment occurred at concentrations that might be therapeutic for humans. We demonstrate that TWPD accurately captures the PD of all drug combinations across the dose-response matrix, improving fit relative to existing models, particularly when matrices show concentration-dependent synergy and antagonism, and can also predict efficacy at untested concentrations. The performance of TWPD is often slightly better in one direction indicating that we can tentatively identify whether a drug is considered upstream or downstream.

## RESULTS

### Two-way pharmacodynamic modeling for the efficacy of drug combinations

To capture concentration-dependent drug-drug interactions and predict the efficacy of drug combinations over the entire dose-response matrix, we developed a two-way pharmacodynamic (TWPD) modeling approach. In a combination composed of drugs 1 and 2, either drug may be assumed to be downstream while the other is assumed to be upstream, where the upstream drug acts independently of the downstream drug but affects the downstream drug activity. The efficacy of a drug combination (*E_combo_*) is the sum of the baseline efficacy contributed by the assumed upstream drug in the absence of the downstream drug ($E_{up|down=0}$) and the assumed downstream drug in the possible presence of the upstream drug ($E_{down|up\geq 0}$). The upstream drug affects not only the PD parameters of the downstream drug but also its maximal efficacy:


(1) $$E_{combo}=E_{down|up\geq 0}+E_{up|down=0}$$

where,


(2) $$E_{up|down=0}=\frac{E_{max,up}\times C_{up}^{n_{up}}}{IC_{50,up}^{n_{up}}+C_{up}^{n_{up}}}$$

(3) $$E_{down|up\geq 0}=\frac{E_{max,\;down}\left(C_{up}\right)\times C_{down}^{n_{down}\left(C_{up}\right)}}{IC_{50,down}^{n_{down}\left(C_{up}\right)}\left(C_{up}\right)+C_{down}^{n_{down}\left(C_{up}\right)}}=\frac{\left(1-E_{up|down=0}\right)\times C_{down}^{n_{down}\left(C_{up}\right)}}{IC_{50,down}^{n_{down}\left(C_{up}\right)}\left(C_{up}\right)+C_{down}^{n_{down}\left(C_{up}\right)}}$$

where *C_down_* and *C_up_* represent the concentrations of the downstream and upstream drug, respectively. The upstream drugs might affect the pharmacodynamic (PD) parameters of the downstream drug, including maximum efficacy (*E_max,down_*), drug concentration associated with 50% inhibition of infection (*IC_50,down_*) and dose response slope (hill coefficient, *n_down_*). The upstream drug therefore influences the dose-response curve shape of the downstream drug in a dose-dependent fashion (28).

**Fig 1:**
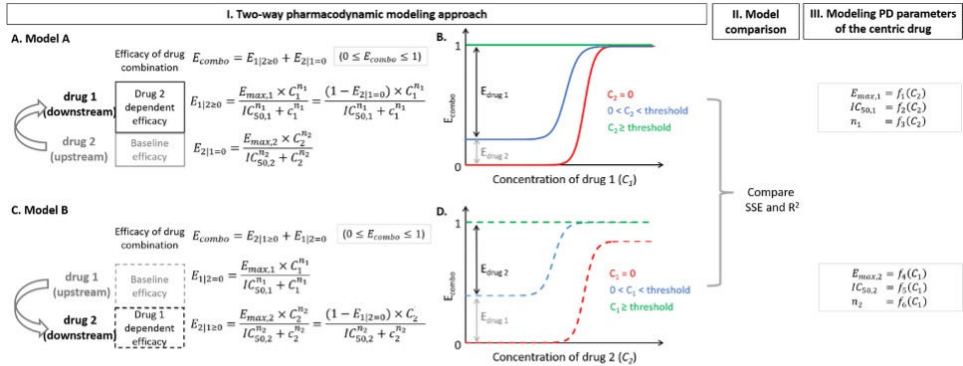
Schematic two-way pharmacodynamic modeling approach for the efficacy of drug combinations. I. A drug combination is composed of drug 1 and drug 2. (**A**) In **Model A**, drug 1 is the assumed downstream drug, and drug 2 is the assumed upstream drug. *E_combo_* equals the sum of the baseline efficacy ($E_{2|1=0}$) provided by drug 2 alone and the efficacy of drug 1 ($E_{1|2\geq 0}$) with drug 2 dependent PD parameters. (**B**) Dose-response curves of the combined efficacy at different concentrations of drug 2 (***C_2_***), as shown in different line colors. Threshold is defined as the concentration of the upstream drug when it provides a baseline efficacy of 1 (100%). (**C**) In **Model B**, drug 1 is assumed to be the upstream drug, and drug 2 is assumed to be the downstream drug. *E_combo_* equals the sum of the baseline efficacy ($E_{1|2=0}$) provided by drug 1 alone and the efficacy of drug 2 ($E_{2|1\geq 0}$) with drug 1 dependent PD parameters. (**D**) Dose-response curves of the combined efficacy at different concentrations of drug 1 (***C_1_***), as shown in different line colors. **II.** Performance of model A and B is compared based on SSE (sum of squared error), R^2^, and AIC with smaller SSE and AIC, and higher R^2^ indicating better model agreement with experimental PD data. **III.** We further modeled the PD parameters of the assumed downstream drug in models A and B as non-mechanistic functions ($f_{i}$) of the concentration of the assumed upstream drug (*C_1_* or *C_2_*) to allow inference of efficacy between tested concentrations.

We call the model above **Model A**; we can also reverse the roles of drugs from downstream to upstream and refer to this as **Model B. Models A** and **B** reflect two competing mechanistic hypotheses for repurposed drugs with unclassified mechanisms in which the true upstream drug is unknown. If the upstream drug is known *a priori* based on known mechanisms of action, then it is sufficient to test **Model A** alone. Thus, the TWPD provides information about the efficacy of a given drug combination and for repurposed drugs with unknown mechanisms of action may inform which drug influences the efficacy of the other (drug 1 → drug 2 or drug 1 ← drug 2), or “directionality” (Fig. 1).

### Two-way PD modeling of possible anti-Ebola drug combinations

**Fig 2:**
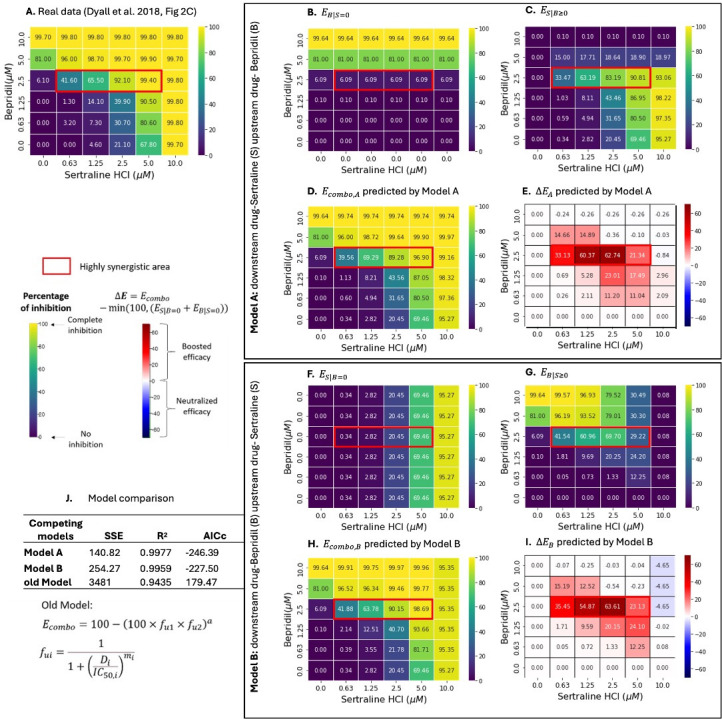
Two-way pharmacodynamic modeling for the efficacy of (sertraline + bepridil) combination against Ebola. (**A**) Empirically quantified *E_combo_* of (sertraline + bepridil) combination in Huh7 cells, data obtained from Dyall et al. (4). In **Model A**, sertraline is the assumed downstream drug, and bepridil is the assumed upstream drug. (**B**) Efficacy of bepridil assuming sertraline is absent ($E_{B|S=0}$). (**C**) Efficacy of sertraline with bepridil-dependent pharmacodynamic parameters ($E_{S|B\geq 0}$). (**D**) Predicted *E_combo_* by model A ($E_{combo,A}=E_{S|B\geq 0}+E_{B|S=0}$). (**E**) Predicted change in *E_combo,A_* ($∆E_{A}=E_{combo,A}-min(100,(E_{S|B=0}+E_{B|S=0}))$ relative to an additive model. In **Model B**, bepridil is the assumed downstream drug, and sertraline is the assumed upstream drug. (**F**) Efficacy of sertraline assuming bepridil is absent ($E_{S|B=0}$). (**G**) Efficacy of bepridil with sertraline-dependent pharmacodynamic parameters ($E_{B|S\geq 0}$). (**H**) Predicted *E_combo_* by model B (${E_{combo,B}=E}_{B|S\geq 0}+E_{S|B=0}$). (**I**) Predicted change in *E_combo,B_* ($∆E_{B}=E_{combo,B}-min(100,(E_{S|B=0}+E_{B|S=0}))$ relative to an additive model. (**J**) Model comparison among Model A, Model B, and our previously published model ($E_{combo}=100-{\left({100\times f}_{u1}\times f_{u2}\right)}^{a}$) (41). Highly synergistic regions, with ∆Bliss values <-0.3, are highlighted in red boxes. The percentage of inhibition ranges from 0 for no inhibition to 100 for complete inhibition of EBOV replication and is shown in the color scheme from purple to yellow. Changes in *E_combo_* (∆E) compared with single drugs are shown in the color scheme from blue to red.

**Fig 3:**
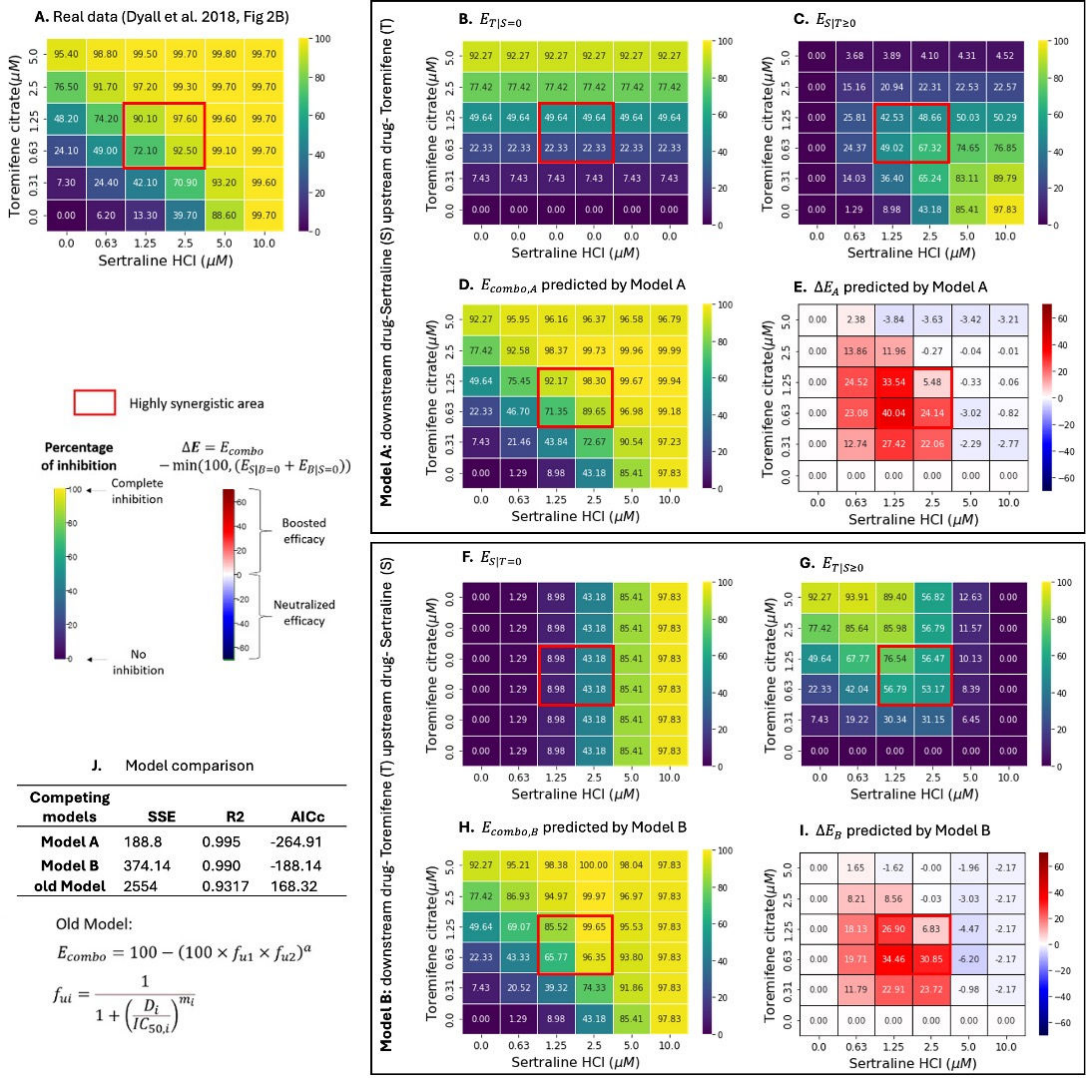
Two-way pharmacodynamic modeling for the efficacy of (sertraline + toremifene) combination against Ebola. (**A**) Empirically quantified *E_combo_* of (sertraline + toremifene) combination in Huh7 cells, data obtained from Dyall et al. (4). In **Model A**, sertraline is the assumed downstream drug, and toremifene is the assumed upstream drug. (**B**) Efficacy of toremifene when sertraline is absent ($E_{T|S=0}$). (**C**) Efficacy of sertraline with toremifene-dependent pharmacodynamic parameters ($E_{S|T\geq 0}$). (**D**) Predicted *E_combo_* by model A ($E_{combo,A}=E_{S|T\geq 0}+E_{T|S=0}$). (**E**) Predicted change in *E_combo,A_* ($∆E_{A}=E_{combo,A}-min(100,(E_{S|T=0}+E_{T|S=0}))$ relative to an additive model. In **Model B**, toremifene is the assumed downstream drug, and sertraline is the assumed upstream drug. (**F**) Efficacy of sertraline when toremifene is absent ($E_{S|T=0}$). (**G**) Efficacy of toremifene with sertraline-dependent pharmacodynamic parameters ($E_{T|S\geq 0}$). (**H**) Predicted *E_combo_* by model B ($E_{combo,B}=E_{T|S\geq 0}+E_{S|T=0}$). (**I**) Predicted change in *E_combo,B_* ($∆E_{B}=E_{combo,B}-\min\left(100,\left(E_{S|T=0}+E_{T|S=0}\right)\right)$ relative to an additive model. (**J**) Model comparison among Model A, Model B and our previously published model ($E_{combo}=100-{\left({100\times f}_{u1}\times f_{u2}\right)}^{a}$) (41). Highly synergistic regions, with ∆Bliss values <-0.3, are highlighted in red boxes. The percentage of inhibition ranges from 0 for no inhibition to 100 for complete inhibition of EBOV replication and is shown in the color scheme from purple to yellow. Changes in *E_combo_* (∆E) compared with single drugs are shown in the color scheme from blue to red.

In a cell culture system, the combination of sertraline + bepridil showed high synergy at 0.63 µM ~ 5 µM sertraline and 2.5 µM of bepridil (Fig. 2A) (4), while sertraline + toremifene were highly synergistic at 1.25 µM ~ 2.5 µM sertraline and 0.63 µM ~ 1.25 µM toremifene (Fig. 3A) (4) against Ebola virus infection. Even at paired concentrations outside of the high synergy zone, drug combinations showed enhanced efficacy beyond what would be predicted by the additive effects of the individual drugs, suggesting multiplicative or synergistic effects across the central portion of the drug concentration matrix.

Using the TWPD model, we fit the dose-response matrix data of (sertraline +bepridil) and (sertraline +toremifene) to **Model A** assuming sertraline as downstream, and bepridil and toremifene as upstream, respectively, each of which provides increasing efficacy with ascending concentrations absent sertraline (Fig. 2B and 3B). Under this model, bepridil optimally enhanced sertraline effect at intermediate concentrations: (i) 0.63 to 2.5 µM bepridil increased efficacy of sertraline, and (ii) levels greater than 2.5 µM bepridil decreased sertraline effect because the single drug effect of bepridil exceeded 80% at 5 µM leaving less room for added sertraline effect (Fig. 2C). Toremifene had a similar effect on low levels of sertraline (≤2.5 µM) and particularly reduced the effect of higher concentrations of sertraline (≥5 µM) (Fig. 3C).

**Model B** f anti-Ebola drug pairs were fit assuming bepridil and toremifene are downstream, respectively, with sertraline as upstream contributing a baseline efficacy (Fig. 2F and 3F). Sertraline had the greatest effects on low to medium levels of bepridil (≤ 5 µM) (Fig. 2G; Fig. S1) but across all concentrations of toremifene (Fig. 3G).

**Model A** and **Model B** outputs closely approximated the experimental data shown in Fig. 2A and 3A). The accuracies of both models exceeded our previously published model (41) (Fig. 2J and 3J), indicating TWPD closely captures dynamics in dose-response matrixes. The model achieved a closer fit to the data than the GPDI model (30) for the sertraline +bepridil pairing but had higher AIC due to a higher number of solved model parameters (Table S1). The model also achieved a closer fit to the data than the GPDI model for the sertraline +toremefine pairing and also had lower AIC score despite more solved model parameters (Table S1). Overall, models A and B, and the GPDI provided excellent fit to both datasets.

**Model A** slightly outperformed **Model B** for bepridil plus sertraline (Fig. 2J), indicating that bepridil is tentatively more likely to be the upstream drug in this pair. **Model A** slightly outperformed **Model B** for toremifene plus sertraline (Fig. 3J), indicating that toremefine is tentatively more likely to be the upstream drug in this pair.

For both anti-Ebola drug combinations, **Models A** and **B** projected high combined efficacies at high concentrations of the assumed downstream and/or upstream drug (Fig. 2D, H, 3D, and H,). Changes in $E_{combo}$ demonstrated maximal increases in combined efficacies in highly synergistic regions with minimal increases at high concentrations of either agent, which already have close to 100% efficacy, limiting any extra possible effect of synergy (Fig. 2E, I, 3E, and I).

### Two-way pharmacodynamic modeling of possible anti-SARS-CoV-2 drug combinations

**Fig 4:**
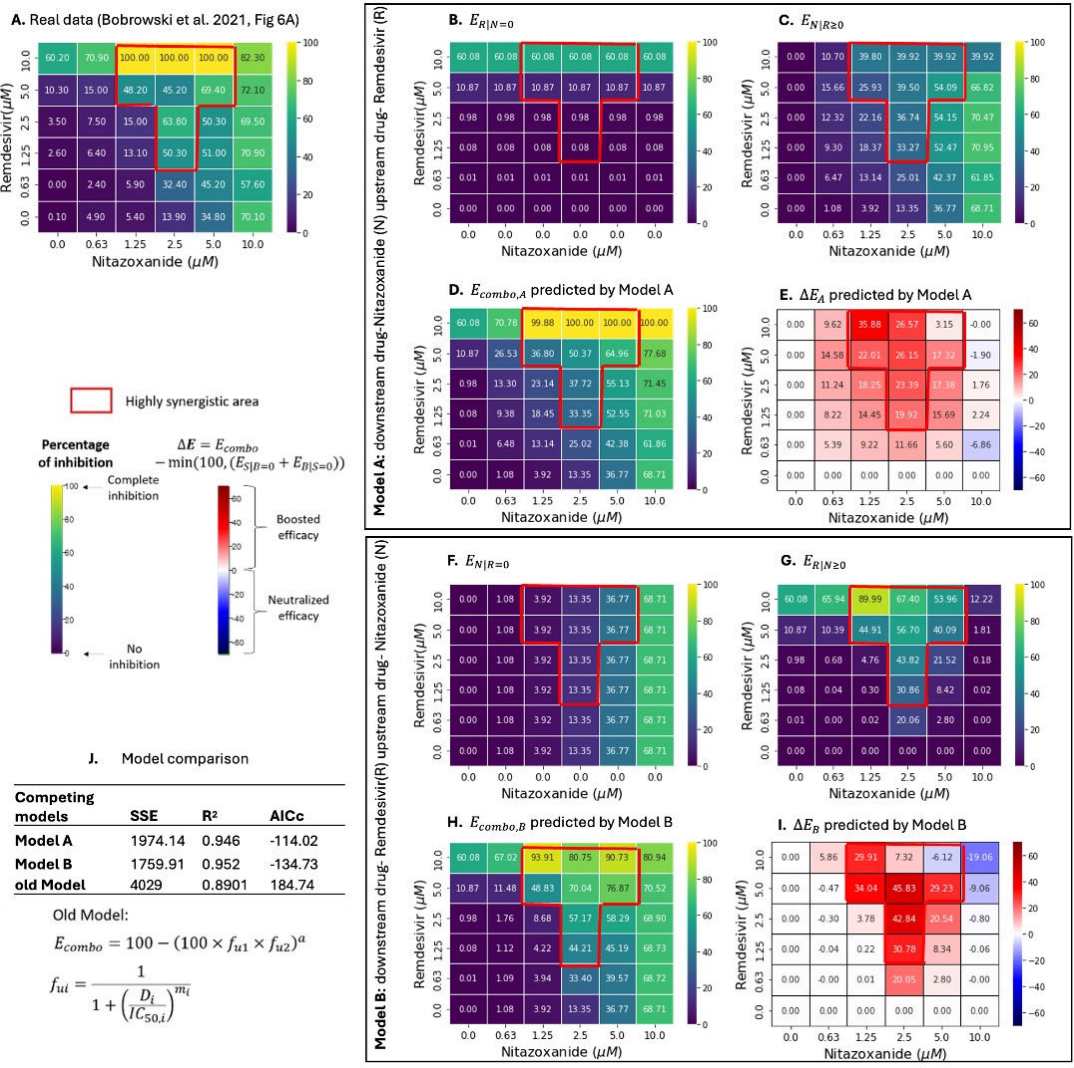
Two-way pharmacodynamic modeling for the efficacy of (nitazoxanide +remdesivir) combination against SARS-CoV-2**. (A**) Empirically quantified *E_combo_* of (nitazoxanide +remdesivir) combination in Vero E6 cells, data obtained from Bobrowski et al. (16). In **Model A**, nitazoxanide is the assumed downstream drug, and remdesivir is the assumed upstream drug. (**B**) Efficacy of remdesivir when nitazoxanide is absent ($E_{R|N=0}$). (**C**) Efficacy of nitazoxanide with remdesivir-dependent pharmacodynamic parameters ($E_{N|R\geq 0}$). (**D**) Predicted *E_combo_* by model A (${E_{combo,A}=E}_{N|R\geq 0}+E_{R|N=0}$). (**E**) Predicted change in *E_combo,A_* ($∆E_{A}=E_{combo,A}-min(100,(E_{N|R=0}+E_{R|N=0}))$ relative to an additive model. In **Model B**, remdesivir is the assumed downstream drug, and nitazoxanide is the assumed upstream drug. (**F**) Efficacy of nitazoxanide when remdesivir is absent ($E_{N|R=0}$). (**G**) Efficacy of remdesivir with nitazoxanide-dependent pharmacodynamic parameters ($E_{R|N\geq 0}$). (**H**) Predicted *E_combo_* by model B ($E_{combo,B}=E_{R|N\geq 0}+E_{N|R=0}$). (**I**) Predicted change in *E_combo,B_* ($∆E_{B}=E_{combo,B}-min(100,(E_{N|R=0}+E_{R|N=0}))$ relative to an additive model. (**J**) Model comparison among Model A, Model B and our previously published model ($E_{combo}=100-{\left({100\times f}_{u1}\times f_{u2}\right)}^{a}$) (41). Highly synergistic regions, with HSA scores < - 0.3, are highlighted in red boxes. The percentage of inhibition ranges from 0 for no inhibition to 100 for complete inhibition of EBOV replication and is shown in the color scheme from purple to yellow. Changes in *E_combo_* (∆E) compared with single drugs are shown in the color scheme from blue to red.

**Fig 5:**
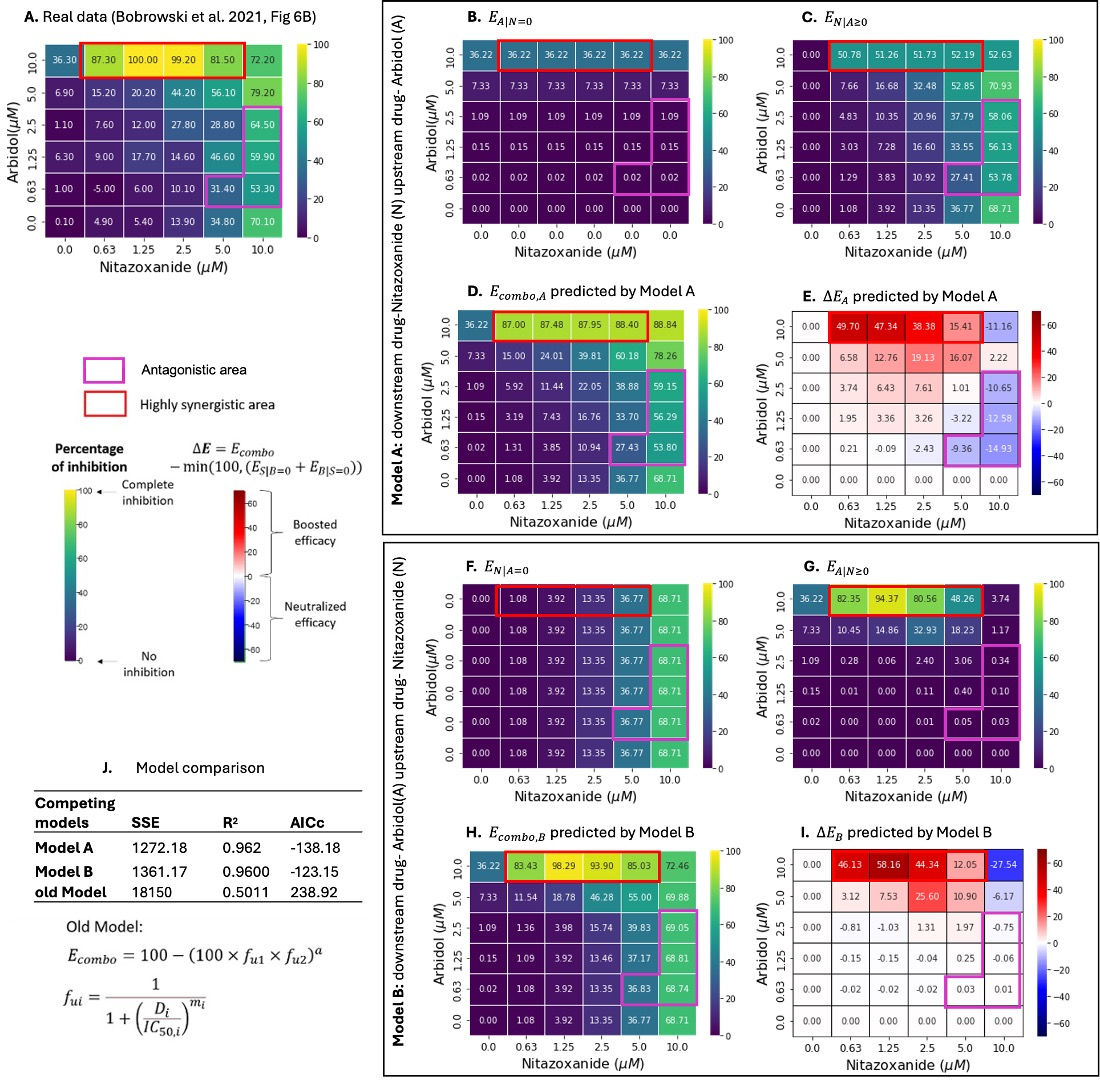
Two-way pharmacodynamic modeling for the efficacy of (nitazoxanide +arbidol) combination against SARS-CoV-2**. (A**) Empirically quantified *E_combo_* of (nitazoxanide +arbidol) combination in Vero E6 cells, data obtained from Bobrowski et al. (16). In **Model A**, nitazoxanide is the assumed downstream drug, and arbidol is the assumed upstream drug. (**B**) Efficacy of arbidol when nitazoxanide is absent ($E_{A|N=0}$). (**C**) Efficacy of nitazoxanide with arbidol dependent pharmacodynamic parameters ($E_{N|A\geq 0}$). (**D**) Predicted *E_combo_* by model A (${E_{combo,A}=E}_{N|A\geq 0}+E_{A|N=0}$). (**E**) Predicted change in *E_combo,A_* ($∆E_{A}=E_{combo,A}-min(100,(E_{N|A=0}+E_{A|N=0}))$ relative to an additive model.In **Model B**, arbidol is the assumed downstream drug, and nitazoxanide is the assumed upstream drug. (**F**) Efficacy of nitazoxanide when arbidol is absent ($E_{N|A=0}$). (**G**) Efficacy of arbidol with nitazoxanide-dependent pharmacodynamic parameters ($E_{A|N\geq 0}$). (**H**) Predicted *E_combo_* by model B (${E_{combo,B}=E}_{A|N\geq 0}+E_{N|A=0}$). (**I**) Predicted change in *E_combo,B_* ($∆E_{B}=E_{combo,B}-min(100,(E_{N|A=0}+E_{A|N=0}))$ relative to an additive model. (**J**) Model comparison among Model A, Model B and our previously published model ($E_{combo}=100-{\left({100\times f}_{u1}\times f_{u2}\right)}^{a}$) (41). Highly synergistic regions, with HSA scores < - 0.3, are highlighted in red boxes. Antagonistic regions, with HAS ≥0.06, are highlighted in purple boxes. The percentage of inhibition ranges from 0 for no inhibition to 100 for complete inhibition of EBOV replication and is shown in the color scheme from purple to yellow. Changes in *E_combo_* (∆E) compared with single drugs are shown in the color scheme from blue to red.

Arbidol and remdesivir demonstrated only medium inhibition of SARS-CoV-2 infection in cell culture when used alone (Fig. 4A and 5A) (16). The addition of nitazoxanide increased the combined efficacy of (nitazoxanide +remdesivir) and (nitazoxanide +arbidol) in highly synergistic regions (Fig. 4A and 5A) (16). Thus, these drug combinations provide a different pattern of data for model fitting.

We modeled the combined efficacy of two possible anti-SARS-CoV-2 drug combinations using TWPD. For **Model A** of (nitazoxanide +remdesivir) and (nitazoxanide +arbidol), nitazoxanide was assumed to be downstream, and the upstream drugs were assumed to be remdesivir and arbidol, respectively. The baseline efficacy provided by either remdesivir or arbidol only reached 60% and 36% efficacy at 10 µM (Fig. 4B and 5B). Remdesivir consistently increased the efficacy of medium levels of nitazoxanide but the effect diminished when both concentrations were high (Fig. 4C). Arbidol at 10 µM was beneficial across a larger range of nitazoxanide (0.625 ~ 5 µM) but was antagonistic to 10 µM nitazoxanide at concentrations between (0.625 ~ 5 µM) (Fig. 5C).

For **Model B** of (nitazoxanide +remdesivir) and (nitazoxanide +arbidol), remdesivir and arbidol were assumed to be downstream in each combination, respectively, with nitazoxanide as the assumed upstream drug providing an increasing baseline efficacy (Fig. 4F and 5F). Nitazoxanide had concentration-dependent effects across all levels of remdesivir (Fig. 4G), while only for medium to high concentrations of arbidol (Fig. 5G). For low concentrations of arbidol, 10 µM nitazoxanide reduced arbidol efficacy (Fig. 5G).

**Model A** and **B** both recapitulated the *in vitro* data (Fig. 4A and 5A) with better accuracies than our previous model (41) (Fig. 4J and 5J). The GPDI model (30) could not achieve parameter convergence with these data sets which included synergy and antagonism within the matrices, highlighting that the TWPD model may be more appropriate for this type of data (Table S1).

**Model B** slightly outperformed **Model A** for nitazoxanide plus remdesivir (Fig. 4J), indicating that nitazoxanide is tentatively more likely to be the upstream drug in this pair. **Model A** slightly outperformed **Model B** for nitazoxanide plus arbidol (Fig. 5J), indicating that nitazoxanide is tentatively more likely to be the upstream drug in this pair. This is compatible with nitazoxanide’s proposed mechanism, which is to block viral entry.*

For both anti-SARS-CoV-2 drug combinations, low to moderate efficacies were common in the dose-response matrixes, as projected by both **Model A** and **Model B** (Fig. 4D, H, 5D, and H). Both models projected great improvements in combined efficacy in highly synergistic areas but diminished effect at high (10 µM) nitazoxanide concentration/antagonistic regions (Fig. 4E, I, 5E, and I).

### Effects of the upstream drug on PD parameters of the downstream drug

By applying TWPD to each drug combination, we identified the potential effects of the upstream drug on the PD parameters of its downstream drug. All predicted parameter values in the various models are listed in Supplementary Tables S2 to S9. A subsequent goal was to identify the mathematical relationship between upstream drug concentration and downstream drug parameters. This allows us to project the combined efficacy of drug pairs at concentrations that were not tested which is critical for projecting therapeutic efficacy when the two drugs are dosed concurrently. It is important to note that we cannot draw mechanistic inferences from these mathematical relationships which capture non-linear correlations between the variables.

**Fig 6:**
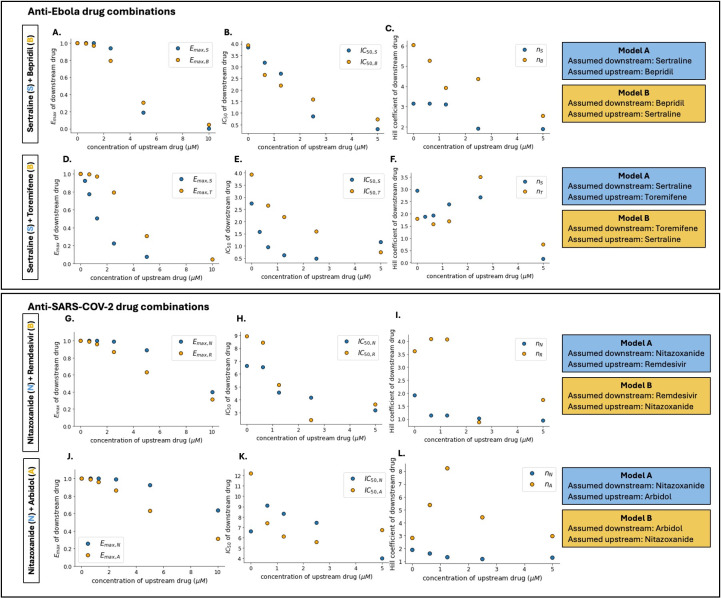
Assumed upstream drug affects the assumed downstream drug’s pharmacodynamic parameters in anti-Ebola and anti-SARS-CoV-2 drug combinations. (**A, B, C**) Estimated *E_max_*, *IC_50_*, and *hill coefficient* of the assumed downstream drug in two-way pharmacodynamic modeling for (sertraline +bepridil), with sertraline (blue dots) as the assumed downstream drug in model A, and bepridil (orange dots) as the assumed downstream drug in model B. (**D, E, F**) Estimated *E_max_*, *IC_50_*, and *hill coefficient* of the assumed downstream drug in two-way pharmacodynamic modeling for (sertraline +toremifene), with sertraline (blue dots) as the assumed downstream drug in model A and toremifene (orange dots) as the assumed downstream drug in model B. (**G, H, I**) Estimated *E_max_*, *IC_50_*, and *hill coefficient* of the assumed downstream drug in two-way pharmacodynamic modeling for (nitazoxanide +remdesivir) with remdesivir (blue dots) as the assumed downstream drug in model A and nitazoxanide (orange dots) as the assumed downstream drug in model B. (**J, K, L**) Estimated *E_max_*, *IC_50_*, and *hill coefficient* of the assumed downstream drug in two-way pharmacodynamic modeling for (nitazoxanide +arbidol), with arbidol (blue dots) as the downstream drug in model A and nitazoxanide (orange dots) as the assumed downstream drug in model B.

In **Model A** of (sertraline +bepridil) for Ebola virus, bepridil decreased the *E_max_*, *IC_50_* and *hill coefficient* of sertraline, with a pattern best captured by an inhibitory sigmoidal function (Fig. 6A through C; Fig. 2A through C). Toremifene reduced the *E_max_* and *IC_50_* of sertraline with a pattern best captured by an inhibitory sigmoidal function and an exponential decay curve, respectively, but only lowered the hill coefficient of sertraline at high concentrations (Fig. 6D through F; Fig. 3A through C). Areas of high potency occurred within ranges of the upstream drug where *E_max_* remained high while *IC_50_* was relatively lowered.

In **Model B** of anti-Ebola drug combinations, sertraline was considered upstream while bepridil or toremifene was assumed to be downstream. Sertraline decreased the *E_max_* and *IC_50_* of bepridil and toremifene, following the pattern of an inhibitory sigmoidal function for *E_max_* and exponential decay for *IC_50_* (Fig. 6A, B, D, and E; Fig. S2D, E, S3D, and E). However, sertraline affected the hill coefficient of bepridil and toremifene in a non-monotonic fashion (Fig. 6C and F; Fig. S2F and 3F).

As with anti-Ebola drug combinations, we observed important impacts of the upstream drug on the downstream drug for anti-SARS-CoV-2 drug combinations, though the assumed upstream drugs in anti-SARS-CoV-2 drug pairs were not as potent alone as those in the anti-Ebola drug combinations. As a result, *E_max_* of the downstream drugs demonstrated less decline for both (nitazoxanide +remdesivir) and (nitazoxanide +arbidol) irrespective of whether **Model A** or **Model B** was applied (Fig. 6G and J; Fig. S4A, D, S5A, and D). In this sense, the synergistic combinatorial effects were more impactful for SARS-CoV-2 than Ebola.

In **Model A** of (nitazoxanide +remdesivir) and (nitazoxanide +arbidol), *IC_50_* of nitazoxanide decayed sigmoidally with remdesivir (Fig. 6H; Fig. S4B), while it and in a non-monotonic pattern with arbidol (Fig. 6K). Remdesivir and arbidol had similar effects on the hill coefficient of nitazoxanide, patterns of which best fit exponential functions (Fig. 6I and L; Fig. S4C and S5C).

In **Model B**, nitazoxanide, the assumed upstream drug, had non-monotonic effects on remdesivir and arbidol. The *IC_50_* of remdesivir decreased exponentially according to nitazoxanide concentration (Fig. 6H; Fig. S4E). Nitazoxanide had an exponential effect on the *IC_50_* of arbidol (Fig. 6K; Fig. S5E). The hill coefficient of remdesivir also changed in a non-monotonic manner with increases and decreases in different ranges of nitazoxanide (Fig. 6I; Fig. S4F), while the hill coefficient of arbidol increased, reaching a peak at 1.25 µM nitazoxanide, and then dropped (Fig. 6L; Fig. S5F).

## DISCUSSION

Nonlinear concentration-dependent drug-drug interactions are observed in high throughput screening of drug combinations. Recapitulating these values with pharmacodynamic modeling is challenging and may limit simulations of treatment trials. We developed a two-way pharmacodynamic modeling (TWPD) approach, by considering the role of a drug in a combination being downstream or upstream and vice versa. Our TWPD model was applied to four previously published antiviral two-drug combinations and captured concentration-dependent drug-drug interactions over the entire experimental dose-response matrix with very high accuracy. For the four pairs, the predictive power of TWPD was somewhat direction dependent, meaning the assumption of whether the drugs act upstream or downstream of each other in the virus lifecycle slightly influenced model performance. Our TWPD model is an efficient modeling approach to predict the pharmacodynamics of drug combinations with concentration-dependent interactions. Because it projects efficacy at experimentally untested but clinically relevant drug concentrations, the TWPD model might be used to predict clinical trial outcomes when combined with pharmacokinetic and intra-host viral dynamic models.

The TWPD modeling approach is an extension of several mechanistic drug synergy models (42–44, 54), the Zero interaction potency (ZIP) (28), and general pharmacodynamic interaction (GPDI) model (30). The ZIP model focuses on diagnosing synergy while GPDI and TWPD capture concentration-dependent drug-drug interactions. All three models consider shifts in the PD parameters of a drug caused by an additional drug if they interact. However, TWPD is more general and makes no *a priori* assumption about which PD parameters might be altered by adding an upstream drug, rather than assuming changes in potency (*IC_50_*) and shape (*hill coefficient*) parameters as with ZIP or in *IC_50_* for competitive interaction and *E_max_* for allosteric interaction as with GPDI. We found that an upstream drug may have complicated impacts on all three parameters including *E_max_*, *IC_50_*, and *hill coefficient*. We compared the best GPDI model and TWPD model for all combinations and demonstrated that GPDI can accurately predict *E_combo_* only when the nature of the interaction across the dose-response matrix of the combination remains consistent. For drug combinations where there are different interactions (i.e., cooperative and antagonism as in the SARS-CoV-2 pairs) in different areas of the dose-response matrix, the GPDI model doesn’t converge or parameters are unidentifiable. Thus, TWPD modeling is flexible, requires little prior knowledge about drug-drug interactions, and has good predictive power.

For the experimental examples we modeled, one or both drugs within a pair were repurposed agents with unknown mechanisms of action. For this reason, we were unable to select the upstream and downstream agents *a priori*. (While arbidol is thought to be a SARS-CoV-2 fusion inhibitor and remdesivir disrupts viral RNA synthesis, nitazoxanide may have various effects at different stages of viral replication.) In cases where the mechanism of action of both drugs is known (for instance, arbidol would likely be upstream of remdesivir), then the upstream drug can be selected prior to modeling and model inferences about concentration-dependent effects of the upstream drug on the pharmacodynamic-parameter values of the downstream drug could be estimated more confidently. To this end, our model has the potential to provide complex mechanistic insights into how drugs with known mechanisms of action interact. One limitation is if the upstream drug and the downstream drug act on the viruses’ lifecycles during overlapping timescales, in which case the downstream drug could in theory impact the upstream drug’s PD parameters as well.

Drug combination screening is mostly conducted for drug pairs. TWPD could also be applied to triple drug combinations by following the logic that a third drug adds additional effects on the PD parameters of the downstream drug if it interacts with the other two drugs by acting on the lifecycle of viruses. The concentration-dependent impacts of two separate upstream drugs on the PD parameters of the downstream drug could be quantified successively. Yet, with *n* multiple drugs in a combination, the number of alternative models follows the rule of permutation and equals *n* factorial (n!), which will grow large with an increase in *n*, and become a limitation of TWPD. Similarly, the number of the interaction terms in the GPDI model and the PD parameters in the ZIP model also increase with more drugs in an interaction. For these reasons, success in modeling PD of triple-drug combinations will need verification against data and will be made substantially easier when applied to drugs with known mechanisms.

For the pairs we modeled, anti-SARS-CoV-2 combinations (nitazoxanide +remdesivir) and (nitazoxanide +arbidol) demonstrated higher potency than anti-Ebola drug combinations (sertraline +bepridil) and (sertraline +toremifene) when drug concentrations were relatively high within the 0 to 10 μM concentration range. Single drug components in the anti-Ebola drug combinations achieved high efficacy when used alone, which might be therapeutically sufficient to inhibit viruses. Drug synergy is more prominent at lower concentrations of anti-Ebola drug pairs than at high concentrations. Single drug components for anti-SARS-CoV-2 drug pairs only have low or moderate efficacy at 10 µM with limited viral inhibition. Combining them with a synergistic agent may allow clinically meaningful improvements.

Several limitations of our model should be noted. Our model is not equipped to predict side effects, or toxic interactions with other drugs, other than toxicity to cell lines which was not observed in our experimental data. Other than possibly identifying the upstream drug, our models do not provide mechanistic insight to the same extent as several existing models (55, 56). Moreover, the selection of drugs for our modeling was based on the comprehensive nature of the drug matrix data for our modeling approach and not on the likelihood of being introduced into the clinic. Since we initiated this work, several effective agents have been licensed for SARS-CoV-2, making our selected agents arbidol and nitazoxanide less relevant for translation to the clinic. Our prior work has also clearly demonstrated that *in vitro* estimates of IC50 often overestimate drug potency in the human body (57–61). Therefore, all modeling of *in vitro* data should be complemented with animal models and human trial treatment data for dose validation. Finally, our modeling is purely PD and is cross-sectional: it is necessary to add a PK component to predict dual drug antiviral efficacy.

With these important caveats in mind, peak and trough concentrations of each component drug in the anti-Ebola and anti-SARS-CoV drug combinations (Table S10) make it plausible that synergy may augment potency during a portion of the dosing interval, though high levels of protein binding may limit the effectiveness of certain agents in humans. Peak and trough concentrations of bepridil are 3.63 (± 1.48) µM and 2.60 (± 1.21) µM (62). Sertraline has higher average peak and trough concentrations in women (C_max_ = 0.54 (± 0.21) µM and C_min_ = 0.35 (± 0.19) µM) than in men (C_max_ = 0.39 (± 0.07) µM and C_min_ = 0.21 (± 0.08) µM) (63). The maximal concentration of toremifene is 1.18 (± 0.42) µM with a trough concentration of 0.29 µM (64). Based on peak and trough concentrations of anti-Ebola drug components, bepridil alone is more effective than sertraline and toremifene with an estimated maximal efficacy of around 81% at C_max_. Combining bepridil with sertraline is more likely to increase combined efficacy compared with sertraline plus toremifene. This is consistent with our previous work in which we combined a drug-drug interaction PD model, pharmacokinetic model for each drug component, and an Ebola intra-host viral dynamics model, and demonstrated a higher potential efficacy when combining 200 mg sertraline with 300 mg bepridil than with 150 mg toremifene under the possible false assumption that the *in vivo IC_50_* in a person equals the experimentally obtained *in vitro IC_50_*.**

For the anti-SARS-CoV-2 drug combinations, peak and trough concentrations are 0.77 (± 0.23) µM and 0.32 (± 0.09) µM for arbidol (65), and 17.34 (± 13.61) and 3.02 (± 7.43) for tizoxanide (a metabolite of nitazoxanide) (66), respectively. Remdesivir reaches peak concentration of 5.34 (± 0.03) µM at 1.03 (± 0.01) hour, and has a short half-life (median t_1/2_ ≈1 hour) without accumulation (67). The combination of (nitazoxanide +remdesivir) may therefore be more likely to increase combined drug efficacy compared with (nitazoxanide +arbidol) because plasma concentrations of arbidol are low with a dosing of 200 mg every 8 hours.

To fully realize the benefits of synergistic repurposed drug combinations, regimens need to be optimized such that the pharmacokinetics of a pair allow the largest proportion of time within the dosing interval at high potency. Our group and others have repeatedly used mathematical modeling as an efficient method to accurately simulate clinical trials: our approach is to combine PD models and pharmacokinetic models (which reflect fluctuations of drug concentrations in humans) with intra-host viral dynamic models which capture the timing and intensity of immune responses (43, 44, 54, 57, 58, 60, 61). By accurately estimating combined efficacy across complex dose-response matrices, our TWPD approach adds the possibility of accurate projections of combination therapies to our clinical trial simulations. By synthesizing TWPD with validated PK and viral dynamic models, it may be possible to optimize the dosing of combination regimens.

## MATERIALS AND METHODS

### Pharmacodynamic data of antiviral drug combinations

PD data of drug combinations are reported in the format of dose-response matrices, where axes are drug concentrations, and each locus represents combined efficacy. Dose-response matrices of anti-Ebola drug combinations, including (sertraline +bepridil) and (sertraline +toremifene), were from Dyall et al. (4). Dose-response matrixes of anti-SARS-CoV2 drug combinations, including (nitazoxanide +remdesivir) and (nitazoxanide +arbidol), were from Bobrowski et al. (16).

### Two-way PD modeling of anti-Ebola and anti-SARS-CoV-2 drug combinations

To model the combined efficacy of anti-Ebola and anti-SARS-CoV-2 drug pairs over the dose-response matrix, we used the two-way pharmacodynamic model: equations (1-3. All code was written in Python (v.3.9.12): https://github.com/sEsmaeili/Combo_Therapy_Modeling. The model assumes efficacy of a combination is the sum of the efficacy of an upstream drug and a downstream drug affected by the upstream one. The model is flexible to switch the roles of drugs in competing mechanistic models **A** and **B**. In **Model A**, we assume drug one is downstream and drug two is upstream. The combined efficacy equals the sum of efficacy provided by single drug 2 ($E_{2|1=0}$) and by drug 1 ($E_{1|2\geq 0}$) with drug 2 dependent PD parameters (Fig. 1A and C). In **Model B**, we assume drug 2 is downstream and drug 1 is upstream. The combined efficacy equals the sum of baseline efficacy of drug 1 ($E_{1|2=0}$) and efficacy provided by drug 2 ($E_{2|1\geq 0}$) with drug 1 dependent PD parameters (Fig. 1B and D).

For each model of a drug combination, we first quantified baseline efficacy of the assumed upstream drug under monotherapy by fitting single efficacy data of the assumed upstream drug to the standard dose-response curve (sigmoid *E_max_* function) (equation 2). At each concentration of the assumed upstream drug, we obtained the efficacy contributed by the assumed downstream drug by subtracting baseline efficacy from observed combined efficacy and further estimated *E_max_*, *IC_50_*, and *hill coefficient* of the assumed downstream drug by fitting to the sigmoid *E_max_* function (equation (3), Fig. S7). Finally, we projected combined efficacy of a pair based on equation (1) (and changes in combined efficacy compared to the sum of single drugs’ efficacies: $∆E=E_{combo}-\min\left(100,\left(E_{1|2=0}+E_{2|1=0}\right)\right).$

The best PD model of combinations was determined by comparing the sum of squared error (SSE), R^2^ , and the corrected AICc of **Model A**, **Model B,** our previous model [$E_{combo}=100-{\left({100\times f}_{u1}\times f_{u2}\right)}^{a}$ , with $f_{ui}=\frac{1}{1+{\left(\frac{D_{i}}{{IC}_{50,i}}\right)}^{m_{i}}}$ , where *D_i_*, *IC_50,i_*, and *m_i_* represent the dose, concentration required for 50% efficacy, hill coefficient of drug *i* while *a* is the power factor aiming to capture synergistic effects in the dose-response matrix (41)], and the published GPDI model. Smaller SSE and AICc, and higher R^2^ indicate better model prediction compared with empirical data set. To calculate the AICc we used the approach in (55),


$$AICc=\sum_{i=1}^{N}\left(n_{i}\ln\frac{SSE}{n_{i}}\right)+2KN(\frac{n_{t}}{n_{t}-KN-N})$$

where N is the number of data sets [for 2WPD, *N*= #upstream concentrations for which we estimate downstream PD parameters + 1 (fitting to the upstream drug alone)], *i* is the index of data set, n_t_ is the total number of data points used in all data sets, and K is the number of model’s free parameters (14 free parameters) plus 1.

Finally, we tried to capture the relationship of the assumed upstream drug concentration on PD parameter values of the assumed downstream drug by using a nonmechanistic modeling approach. Specifically, for a PD parameter of a downstream drug, we fitted several types of models (exponential, polynomial linear, and sigmoidal) between the concentration of the assumed upstream drug and the PD parameter, to mathematically capture the relationship between the two without trying to understand the mechanism of drug-drug interaction.

#### Biosafety

We did not perform experiments with any select agent.

## SUPPLEMENTAL MATERIAL

Supplemental material aac.01015-23-s0001.docx Figures S1 to S7; Tables S1 to S10.

ASM does not own the copyrights to Supplemental Material that may be linked to, or accessed through, an article. The authors have granted ASM a non-exclusive, world-wide license to publish the Supplemental Material files. Please contact the corresponding author directly for reuse.
