# Supplementary material for: Two-way pharmacodynamic modeling of drug combinations and its application to pairs of repurposed Ebola and SARS-CoV-2 agents
Source: Antimicrob Agents Chemother. 2024 Mar 12;68(4):e01015-23. doi: 10.1128/aac.01015-23 (PMC10989026; doi:10.1128/aac.01015-23)
Supplement: Supplemental material — Figures S1 to S7; Tables S1 to S10. [file aac.01015-23-s0001.docx]

**Supplementary Figures**

**
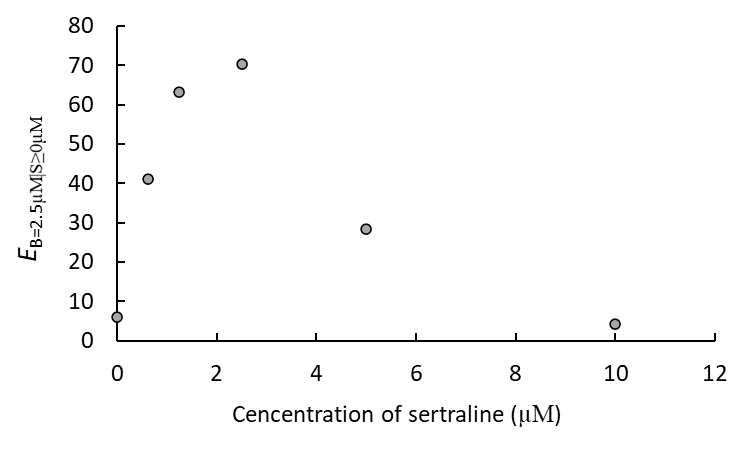
**

**Supplementary Figure 1. Non-monotonic effects of sertraline on the efficacy of 2.5 μM bepridil.**


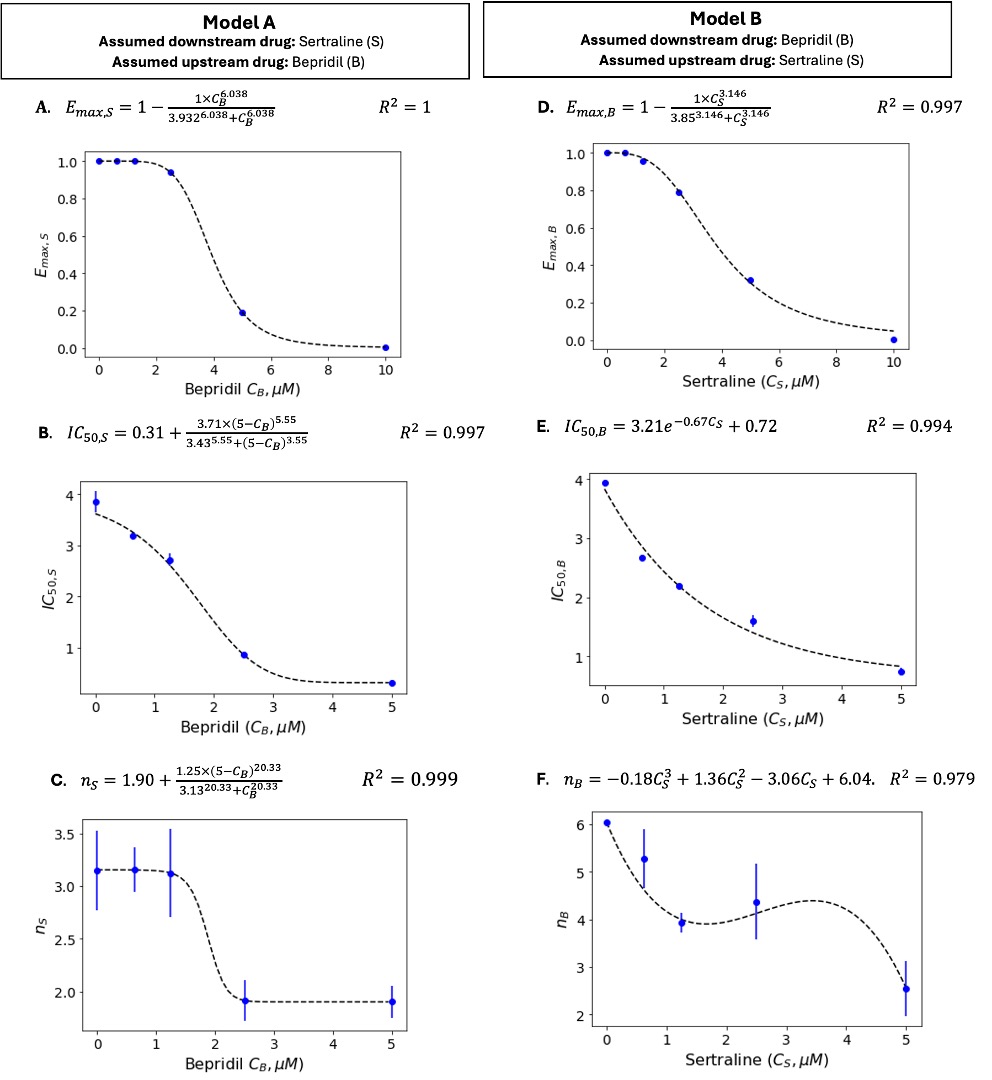


**Supplementary Figure 2. Non-mechanistic modeling of the effects of the assumed upstream drug on pharmacodynamic (PD) parameters of the assumed downstream drug for (sertraline + bepridil).** Solid circle represents estimated PD parameters of the assume downstream drug in the two-way pharmacodynamic modeling of (sertraline + bepridil). Non-mechanistic functions are provided, and function projections are indicated in dashed lines.

**
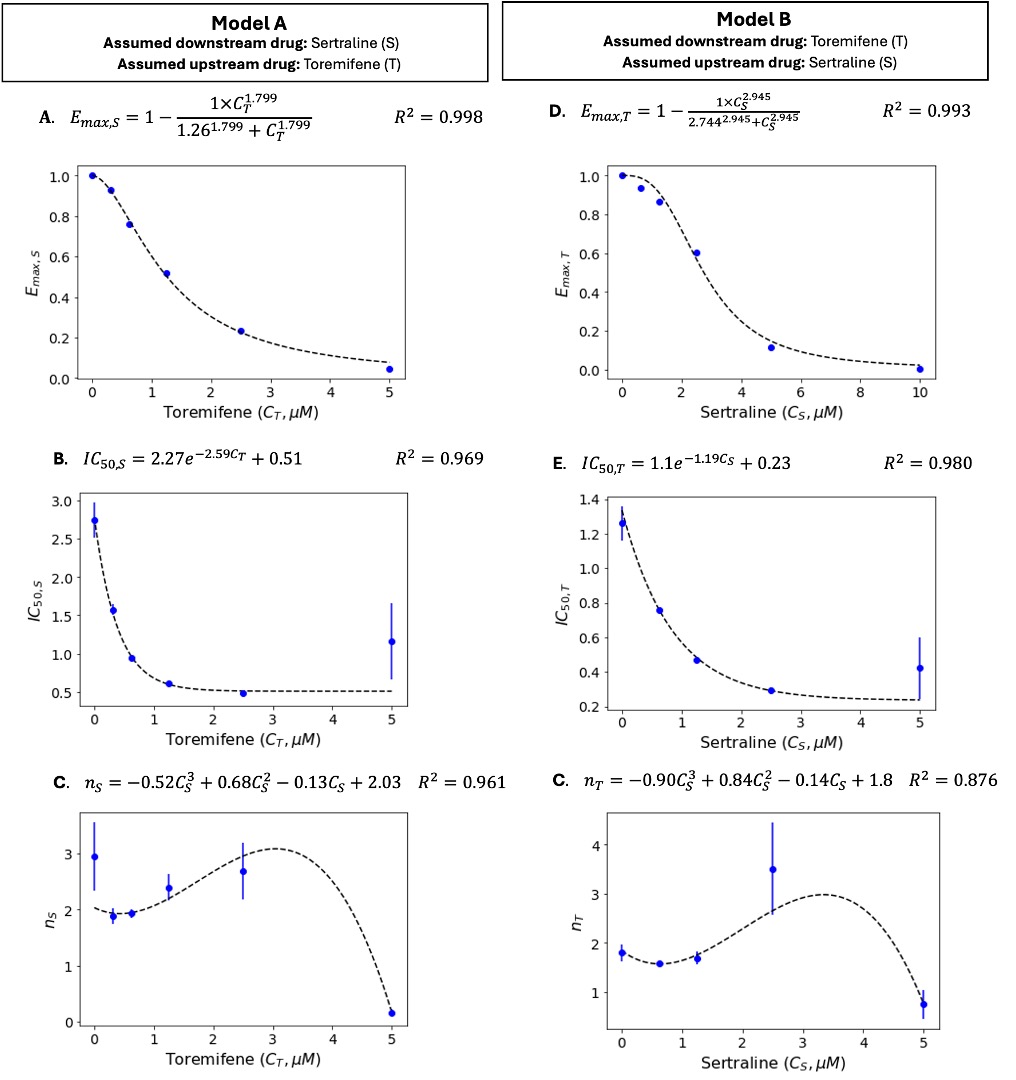
**

**Supplementary Figure 3.** **Non-mechanistic modeling of the effects of the assumed upstream drug on pharmacodynamic (PD) parameters of the assumed downstream drug for (sertraline + toremifene).** Solid circle represents estimated PD parameters of the assumed downstream drug in the two-way pharmacodynamic modeling of (sertraline + toremifene). Non-mechanistic functions are provided, and function projections are indicated in dashed lines.

**
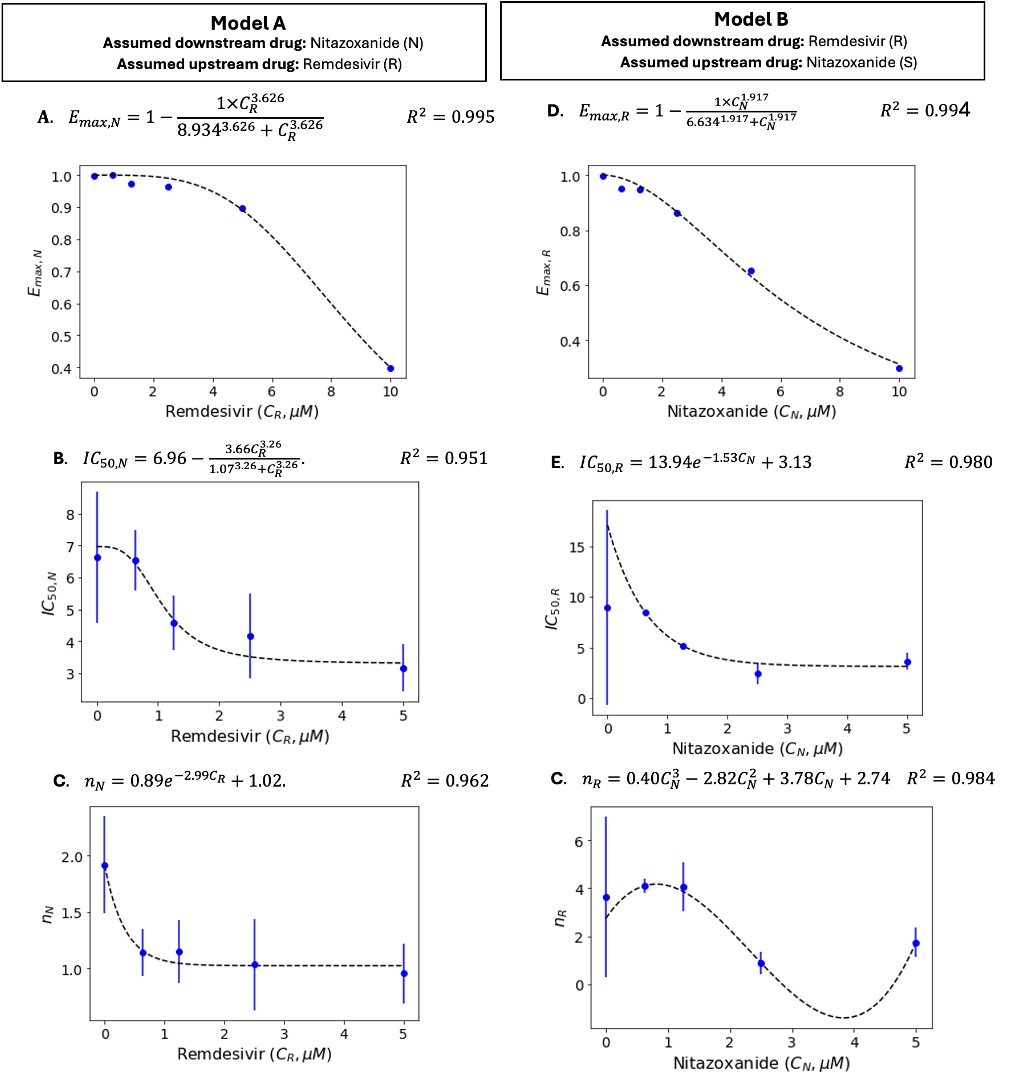
Supplementary Figure 4.** **Non-mechanistic modeling of the effects of the assumed upstream drug on pharmacodynamic (PD) parameters of the assumed downstream drug for (nitazoxanide + remdesivir).** Solid circle represents estimated PD parameters of the assumed downstream drug in the two-way pharmacodynamic modeling of (nitazoxanide + remdesivir). Non-mechanistic functions are provided, and function projections are indicated in dashed lines.


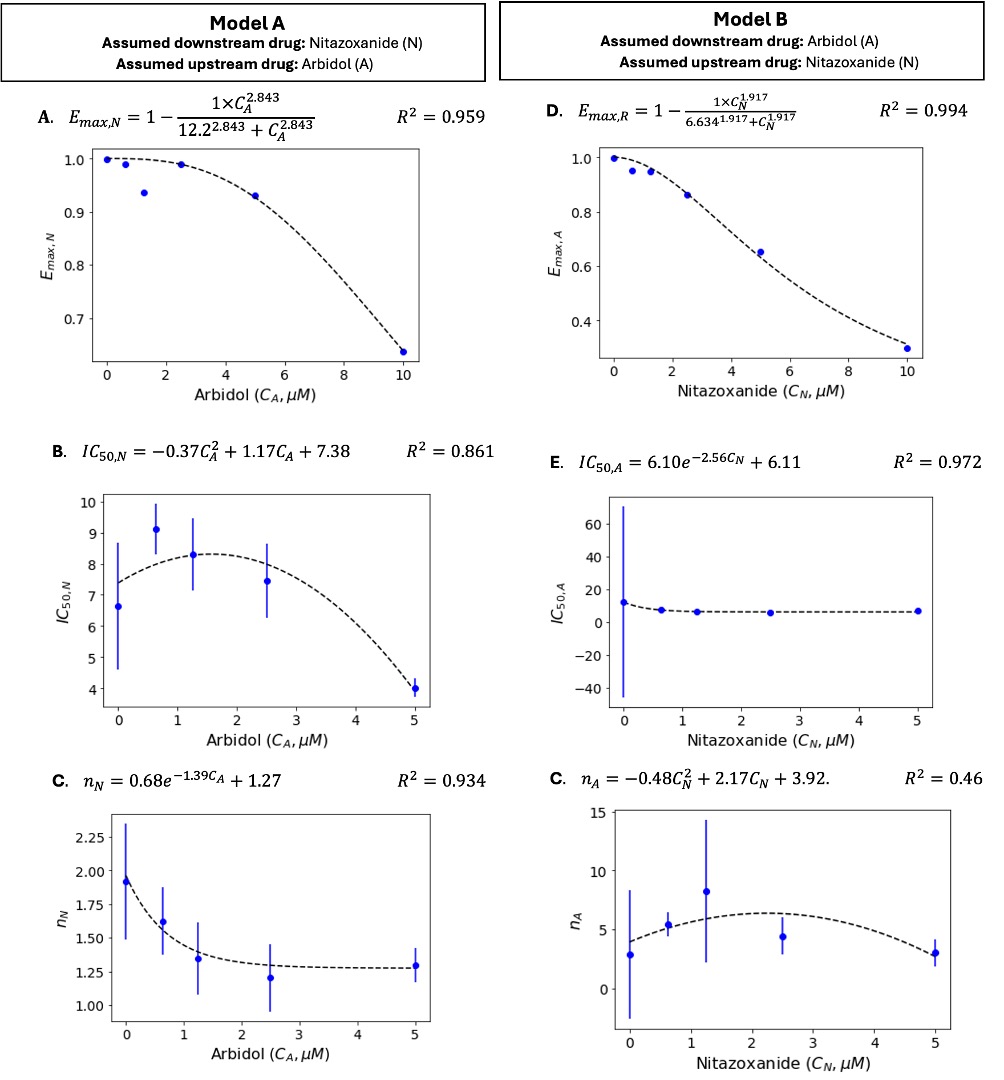


**Supplementary Figure 5.** **Non-mechanistic modeling of the effects of the assumed upstream drug on pharmacodynamic (PD) parameters of the assumed downstream drug for (nitazoxanide + arbidol).** Solid circle represents estimated PD parameters of the assumed downstream drug in the two-way pharmacodynamic modeling of (nitazoxanide + arbidol). Non-mechanistic functions are provided, and function projections are indicated in dashed lines.

**
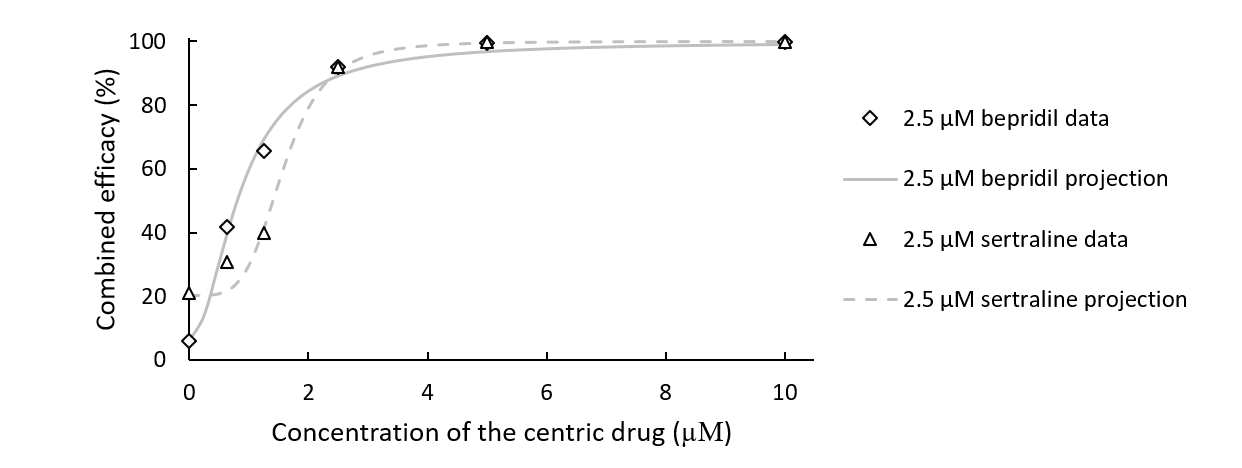
**

**Supplementary Figure 6. Examples of combined efficacy at 2.5 µM assumed upstream drug for (sertraline + bepridil).** When sertraline is the assumed downstream drug, bepridil provides a baseline efficacy of 6.10%. Empirical data and model A projection of the combined efficacy of sertraline and 2.5 µM bepridil are shown in diamonds and a solid gray line respectively. When bepridil is the assumed downstream drug, sertraline provides a baseline efficacy of 20.45%. Empirical data and model B projection of the combined efficacy of bepridil and 2.5 µM sertraline are shown in triangles and a dashed gray line accordingly.

**
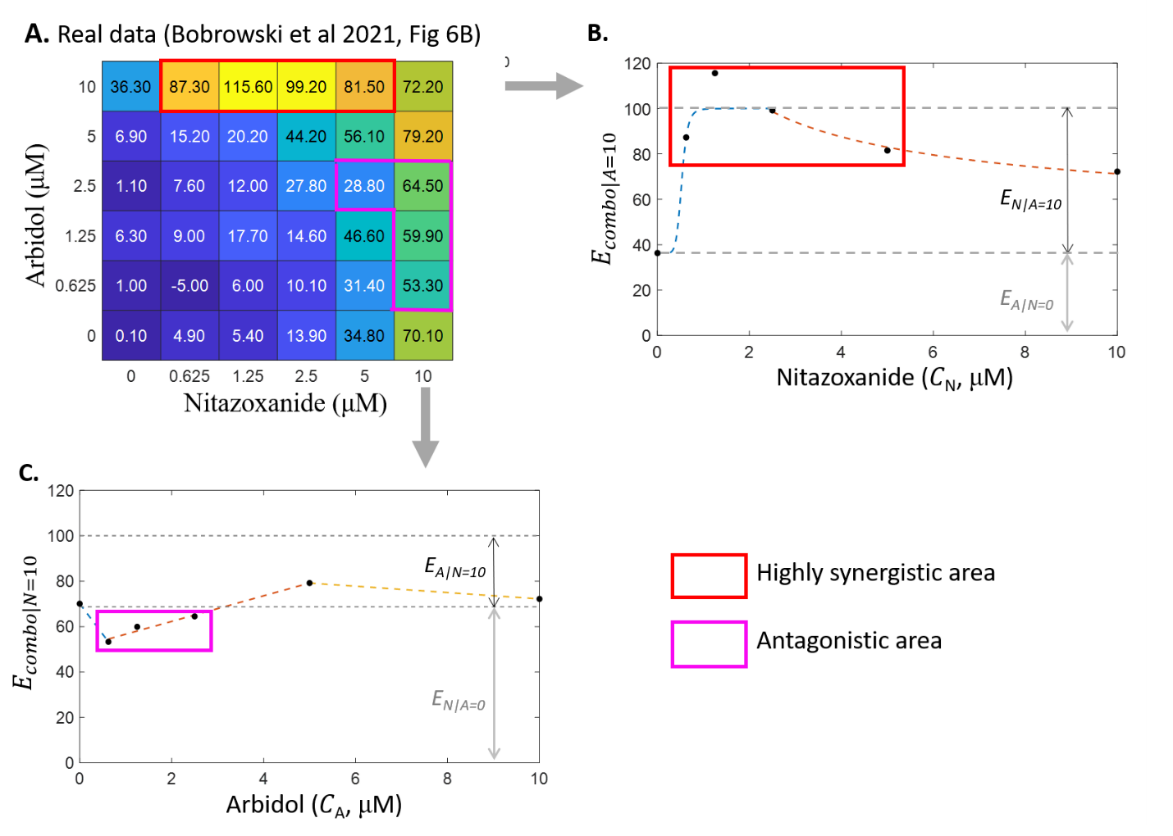
**

**Supplementary Figure 7. Piece-wise data fitting to the combined efficacy of (nitazoxanide + arbidol) to capture decreases in *E_combo_* caused by synergy or antagonism of nitazoxanide.** Highly synergistic regions, with HSA scores < - 0.3, are highlighted in red boxes. Antagonistic regions, with HAS ≥ 0.06, are highlighted in purple boxes. A) *E_combo_* of arbidol and nitazoxanide at all concentration combinations. (1) B) and C), black dots are measured *E_combo_* at 10 µM arbidol (*E_combo|A=10_*) and *E_combo_* at 10 µM nitazoxanide (*E_combo|N=10_*), respectively. Colors of dashed line represent piece-wise model predictions of *E_combo|A=10_* and *E_combo|N=10_*, calculated as the summation of a based line efficacy (gray) and the efficacy of the assumed downstream drug (nitazoxanide for B and arbidol for C).

**Supplementary Tables**

**Supplementary Table 1.** **General pharmacodynamic interaction (GPDI) Model Development all combinations.**

**Assumes interaction on EC_50_ (equations below copied from Wicha et al. ^3^)**

**GPDI_EC50:**

$E_{A}=\frac{\left( Emax_{A} C_{A}^{H_{A}} \right)}{\left( EC{50}_{A} \left( 1 +\frac{{Int_{AB} C}_{B}}{EC{50}_{Int_{AB}}+ C_{B}} \right) \right)^{H_{A}} + C_{A}^{H_{A}}}$, $E_{B}=\frac{\left( Emax_{B} C_{B}^{H_{B}} \right)}{\left( EC{50}_{B} \left( 1 +\frac{Int_{BA} C_{A}}{EC{50}_{Int_{BA}}+ C_{A}} \right) \right)^{H_{B}} + C_{B}^{H_{B}}}$

**GPDI_EC50_Int:**

$E_{A}=\frac{\left( Emax_{A} C_{A}^{H_{A}} \right)}{\left( EC{50}_{A} \left( 1 +\frac{{Int C}_{B}}{EC{50}_{A}+ C_{B}} \right) \right)^{H_{A}} + C_{A}^{H_{A}}}$, $E_{B}=\frac{\left( Emax_{B} C_{B}^{H_{B}} \right)}{\left( EC{50}_{B} \left( 1 +\frac{Int C_{A}}{EC{50}_{B}+ C_{A}} \right) \right)^{H_{B}} + C_{B}^{H_{B}}}$

**GPDI_EC50_reduced2:**

$E_{A}=\frac{\left( Emax_{A} C_{A}^{H_{A}} \right)}{\left( EC{50}_{A} \left( 1 +\frac{{Int C}_{B}}{EC{50}_{Int_{AB}}+ C_{B}} \right) \right)^{H_{A}} + C_{A}^{H_{A}}}$, $E_{B}=\frac{\left( Emax_{B} C_{B}^{H_{B}} \right)}{\left( EC{50}_{B} \left( 1 +\frac{Int C_{A}}{EC{50}_{Int_{BA}}+ C_{A}} \right) \right)^{H_{B}} + C_{B}^{H_{B}}}$

**GPDI_EC50_reduced3:**

$E_{A}=\frac{\left( Emax_{A} C_{A}^{H_{A}} \right)}{\left( EC{50}_{A} \left( 1 +\frac{{Int_{AB} C}_{B}}{EC{50}_{A}+ C_{B}} \right) \right)^{H_{A}} + C_{A}^{H_{A}}}$, $E_{B}=\frac{\left( Emax_{B} C_{B}^{H_{B}} \right)}{\left( EC{50}_{B} \left( 1 +\frac{Int_{BA} C_{A}}{EC{50}_{B}+ C_{A}} \right) \right)^{H_{B}} + C_{B}^{H_{B}}}$

**GPDI_EC50_reduced4:**

$E_{A}=\frac{\left( Emax_{A} C_{A}^{H_{A}} \right)}{\left( EC{50}_{A} \left( 1 +\frac{C_{B}}{EC{50}_{Int_{AB}}+ C_{B}} \right) \right)^{H_{A}} + C_{A}^{H_{A}}}$, $E_{B}=\frac{\left( Emax_{B} C_{B}^{H_{B}} \right)}{\left( EC{50}_{B} \left( 1 +\frac{C_{A}}{EC{50}_{Int_{BA}}+ C_{A}} \right) \right)^{H_{B}} + C_{B}^{H_{B}}}$

$$E_{comb} = E_{A} + E_{B} - E_{A} \times E_{B}$$

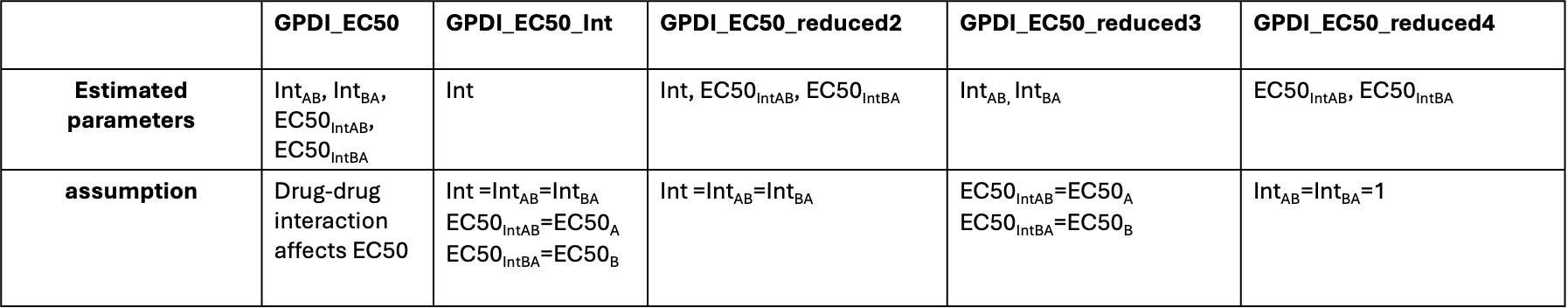


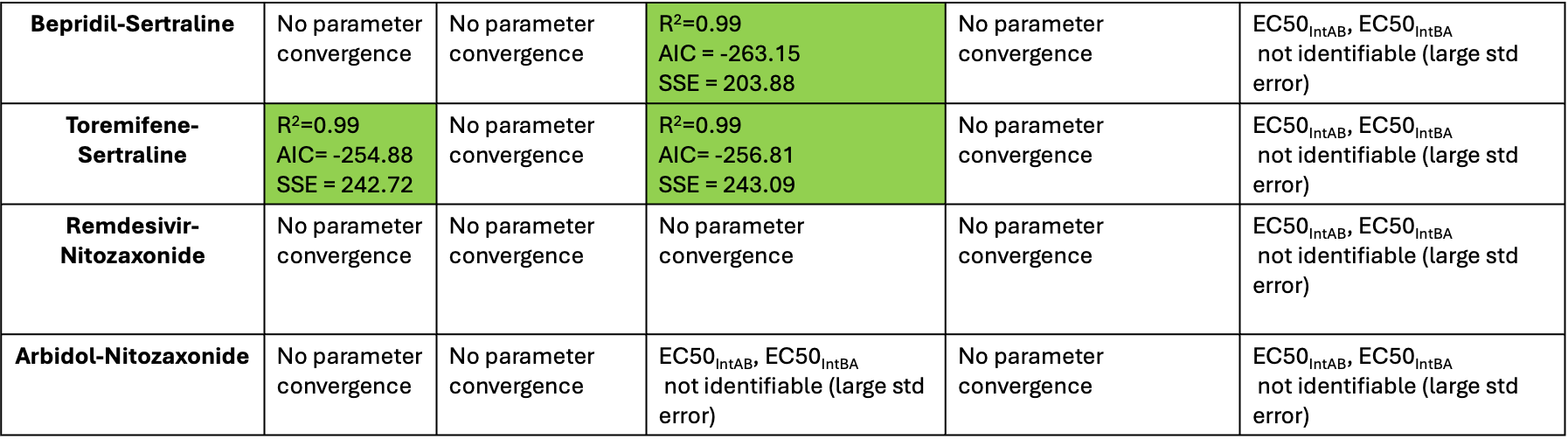


**Supplementary Table 2.** **Estimated PD parameters of sertraline (S, assumed downstream drug) at different levels of bepridil (B, assumed upstream drug).**

| Bepridil (μM) | E_max, S_ | IC_50, S_ | n_S_ | SSE | R^2^ |
| --- | --- | --- | --- | --- | --- |
| 0 | 1 | 3.85 | 3.146 | 0.0026 | 0.9970 |
| 0.63 | 1 | 3.190 | 3.157 | 0.0019 | 0.9979 |
| 1.25 | 0.999 | 2.718 | 3.124 | 0.0064 | 0.9935 |
| 2.5 | 0.939 | 0.8577 | 1.916 | 0.0033 | 0.9953 |
| 5 | 0.1899 | 0.314 | 1.902 | 7.75E-06 | 0.9997 |
| 10 | 0.0036 | Parameters not identifiable | | | |

**Supplementary Table 3.** **Estimated PD parameters of bepridil (B, assumed downstream drug) at different levels of sertraline (S, assumed upstream drug).**

| Sertraline (μM) | E_max, B_ | IC_50, B_ | n_B_ | SSE | R^2^ |
| --- | --- | --- | --- | --- | --- |
| 0 | 1 | 3.932 | 6.038 | 1.287e-6 | 0.9999 |
| 0.63 | 0.9966 | 2.664 | 5.27 | 0.0010 | 0.9990 |
| 1.25 | 0.9718 | 2.189 | 3.925 | 0.000725 | 0.999 |
| 2.5 | 0.7955 | 1.598 | 4.373 | 0.00729 | 0.989 |
| 5 | 0.3053 | 0.737 | 2.541 | 0.00137 | 0.984 |
| 10 | 0.0473 | Parameters not identifiable | | | |

**Supplementary Table 4. Estimated PD parameters of toremifene (T, assumed downstream drug) at different levels of sertraline (S, assumed upstream drug).**

| Sertraline (μM) | E_max, T_ | IC_50, T_ | n_T_ | SSE | R^2^ |
| --- | --- | --- | --- | --- | --- |
| 0 | 1 | 1.26 | 1.799 | 0.00159 | 0.998 |
| 0.63 | 0.987 | 0.76 | 1.58 | 0.00034 | 0.999 |
| 1.25 | 0.9102 | 0.467 | 1.69 | 0.0021 | 0.9965 |
| 2.5 | 0.5681 | 0.293 | 3.5 | 0.0020 | 0.9929 |
| 5 | 0.1459 | 0.422 | 0.753 | 0.0011 | 0.901 |
| 10 | 0.0217 | Parameters not identifiable | | | |

**Supplementary Table 5.** **Estimated PD parameters of sertraline (S, assumed downstream drug) at different levels of toremifene (T, assumed upstream drug).**

| Toremifene (μM) | E_max, S_ | IC_50, S_ | n_S_ | SSE | R^2^ |
| --- | --- | --- | --- | --- | --- |
| 0 | 1 | 2.744 | 2.945 | 0.00685 | 0.992 |
| 0.31 | 0.9257 | 1.574 | 1.880 | 0.00287 | 0.996~~1~~ |
| 0.63 | 0.7768 | 0.946 | 1.926 | 0.000419 | 0.9999 |
| 1.25 | 0.5036 | 0.617 | 2.396 | 0.000432 | 0.9979 |
| 2.5 | 0.2257 | 0.483 | 2.681 | 0.000115 | 0.9972 |
| 5 | 0.0773 | 1.160 | 0.159 | 2.18e-5 | 0.9851 |

**Supplementary Table 6.** **Estimated PD parameters of remdesivir (R, assumed downstream drug) at different levels of nitazoxanide (N, assumed upstream drug).**

| Nitazoxanide (μM) | E_max, R_ | IC_50, R_ | n_R_ | SSE | R^2^ |
| --- | --- | --- | --- | --- | --- |
| 0 | 1 | 8.934 | 3.626 | 0.001306 | 0.9953 |
| 0.625 | 0.9893 | 8.44 | 4.089 | 0.00122 | 0.996 |
| 1.25 | 0.9608 | 5.163 | 4.073 | 0.0104 | 0.985 |
| 2.5 | 0.8666 | 2.436 | 0.887 | 0.106 | 0.754 |
| 5 | 0.6323 | 3.650 | 1.748 | 0.0311 | 0.885 |
| 10 | 0.3129 | Parameters not identifiable | | | |

**Supplementary Table 7.** **Estimated PD parameters of nitazoxanide (N, assumed downstream drug) at different levels of remdesivir (R, assumed upstream drug).**

| Remdesivir (μM) | E_max, N_ | IC_50, N_ | n_N_ | SSE | R^2^ |
| --- | --- | --- | --- | --- | --- |
| 0 | 6.634 | 1.917 | 0.002301 | 0.9936 | 6.634 |
| 0.625 | 6.546 | 1.141 | 0.01497 | 0.9504 | 6.546 |
| 1.25 | 4.581 | 1.147 | 0.0324 | 0.9206 | 4.581 |
| 2.5 | 4.168 | 1.032 | 0.0812 | 0.8155 | 4.168 |
| 5 | 3.174 | 0.956 | 0.0335 | 0.9023 | 3.174 |
| 10 | 0.3992 | Parameters not identifiable | | | |

**Supplementary Table 8.** **Estimated PD parameters of arbidol (A, assumed downstream drug) at different levels of nitazoxanide (N, assumed upstream drug).**

| Nitazoxanide (μM) | E_max, A_ | IC_50, A_ | n_A_ | SSE | R^2^ |
| --- | --- | --- | --- | --- | --- |
| 0 | 1 | 12.2 | 2.843 | 0.003894 | 0.9595 |
| 0.625 | 0.9893 | 7.43 | 5.395 | 0.012 | 0.9787 |
| 1.25 | 0.9608 | 6.145 | 8.23 | 0.0194 | 0.97 |
| 2.5 | 0.8664 | 5.58 | 4.43 | 0.0176 | 0.969 |
| 5 | 0.632 | 6.76 | 2.99 | 0.0236 | 0.882 |
| 10 | 0.3129 | Parameters not identifiable | | | |

**Supplementary Table 9.** **Estimated PD parameters of nitazoxanide (N, assumed downstream drug) at different levels of arbidol (A, assumed upstream drug).**

| Arbidol (μM) | E_max, R_ | IC_50, R_ | n_R_ | SSE | R^2^ |
| --- | --- | --- | --- | --- | --- |
| 0 | 1 | 6.634 | 1.917 | 0.002301 | 0.9936 |
| 0.625 | 0.9998 | 9.106 | 1.623 | 0.00689 | 0.972 |
| 1.25 | 0.9985 | 8.301 | 1.34 | 0.0138 | 0.944 |
| 2.5 | 0.9891 | 7.46 | 1.2 | 0.0166 | 0.936 |
| 5 | 0.9266 | 4.021 | 1.298 | 0.0050 | 0.9869 |
| 10 | 0.6378 | Model not converging | | | |

**Supplementary Table 10.** **Peak and trough concentrations of drug components in anti-Ebola and anti-SARS-CoV-2 drug combinations.**

| **Drug** | **Dosing** | **Participant’s age** | **C_max_** (µM)  Mean (SD) | **C_min_** (µM)  Mean (SD) | **Protein binding** |
| --- | --- | --- | --- | --- | --- |
| Bepridil (2) | 100 mg BID for 21 days | 79 ± 5 | 3.63 (1.48) | 2.60 (1.21) | 99.8% (3) |
| Sertraline (4) | 50 mg SID for 3 days, 100 mg SID for 3 days, 150 mg SID for 3 days, 200 mg SID for the remaining 21 days | 18 ~ 48 | M: 0.39 (0.07)  F: 0.54 (0.21) | M: 0.21 (0.08)  F: 0.35 (0.19) | 98% (5) |
| Toremifene (6) | 100 mg SID for 12 weeks | 55 ~ 75 female | 1.18 (0.42) | 0.29 | >95% (7) |
| Arbidol (8) | 200 mg TID for 7 days | 18 ~ 35 | 0.77 (0.23) | 0.32 (0.09) | 90% (9) |
| Tizoxanide (10) | 600 mg BID for 5 days | 12 ~ 65 | 17.34 (13.61) | 3.02 (7.43) | >99.9% (11) |
| Remdesivir (12) | 150 mg i.v. over a 1-hour period/day for 7 days in cohort 1 and 14 days in cohort 2. | 19 ~ 55 | 5.34 (0.03) | Not detected | 80-90% (13) |

SID: Once daily; BID: Twice daily; TID: Three times a day. i.v.: intravenous route.

**Reference**

1. Bobrowski T, Chen L, Eastman RT, Itkin Z, Shinn P, Chen CZ, Guo H, Zheng W, Michael S, Simeonov A, Hall MD, Zakharov AV, Muratov EN. 2021. Synergistic and Antagonistic Drug Combinations against SARS-CoV-2. Mol Ther 29:873-885.

2. Benet LZ. 1985. Pharmacokinetics and metabolism of bepridil. Am J Cardiol 55:8C-13C.

3. Pritchard JF, McKown LA, Dvorchik BH, O'Neill PJ. 1985. Plasma protein binding of bepridil. J Clin Pharmacol 25:347-53.

4. Ronfeld RA, Tremaine LM, Wilner KD. 1997. Pharmacokinetics of sertraline and its N-demethyl metabolite in elderly and young male and female volunteers. Clin Pharmacokinet 32 Suppl 1:22-30.

5. Huddart R, Hicks JK, Ramsey LB, Strawn JR, Smith DM, Bobonis Babilonia M, Altman RB, Klein TE. 2020. PharmGKB summary: sertraline pathway, pharmacokinetics. Pharmacogenet Genomics 30:26-33.

6. DeGregorio MW, Wurz GT, Taras TL, Erkkola RU, Halonen KH, Huupponen RK. 2000. Pharmacokinetics of (deaminohydroxy)toremifene in humans: a new, selective estrogen-receptor modulator. Eur J Clin Pharmacol 56:469-75.

7. Taras TL, Wurz GT, Linares GR, DeGregorio MW. 2000. Clinical pharmacokinetics of toremifene. Clin Pharmacokinet 39:327-34.

8. Sun Y, He X, Qiu F, Zhu X, Zhao M, Li-Ling J, Su X, Zhao L. 2013. Pharmacokinetics of single and multiple oral doses of arbidol in healthy Chinese volunteers. Int J Clin Pharmacol Ther 51:423-32.

9. Haviernik J, Štefánik M, Fojtíková M, Kali S, Tordo N, Rudolf I, Hubálek Z, Eyer L, Ruzek D. 2018. Arbidol (Umifenovir): A Broad-Spectrum Antiviral Drug That Inhibits Medically Important Arthropod-Borne Flaviviruses. Viruses 10.

10. Haffizulla J, Hartman A, Hoppers M, Resnick H, Samudrala S, Ginocchio C, Bardin M, Rossignol JF, Group UNICS. 2014. Effect of nitazoxanide in adults and adolescents with acute uncomplicated influenza: a double-blind, randomised, placebo-controlled, phase 2b/3 trial. Lancet Infect Dis 14:609-18.

11. Stockis A, Allemon AM, De Bruyn S, Gengler C. 2002. Nitazoxanide pharmacokinetics and tolerability in man using single ascending oral doses. Int J Clin Pharmacol Ther 40:213-20.

12. Humeniuk R, Mathias A, Cao H, Osinusi A, Shen G, Chng E, Ling J, Vu A, German P. 2020. Safety, Tolerability, and Pharmacokinetics of Remdesivir, An Antiviral for Treatment of COVID-19, in Healthy Subjects. Clin Transl Sci 13:896-906.

13. Tempestilli M, Caputi P, Avataneo V, Notari S, Forini O, Scorzolini L, Marchioni L, Ascoli Bartoli T, Castilletti C, Lalle E, Capobianchi MR, Nicastri E, D'Avolio A, Ippolito G, Agrati C, Group CIS. 2020. Pharmacokinetics of remdesivir and GS-441524 in two critically ill patients who recovered from COVID-19. J Antimicrob Chemother 75:2977-2980.
